# Changes in the position and inclination of the upper incisor and upper lip post-orthodontic treatment: case reports

**DOI:** 10.21142/2523-2754-1103-2023-170

**Published:** 2023-09-26

**Authors:** Alicia Chacón Moreno, Nelly Huasco Huarcaya

**Affiliations:** Orthodontics Division, Stomatology Career, Universidad Científica del Sur. Lima, Perú. ali_ch3@hotmail.com, lozanoh77@hotmail.com Universidad Científica del Sur Orthodontics Division Stomatology Career Universidad Científica del Sur Lima Peru ali_ch3@hotmail.com lozanoh77@hotmail.com

**Keywords:** upper incisor, upper lip, soft tissue, tooth movement, orthodontics, incisivo superior, labio superior, tejidos blandos, movimiento dentario, ortodoncia

## Abstract

**Objective:**

To determine the changes in the position and inclination of the upper incisor and upper lip after orthodontic treatment in a series of 3 clinical cases.

**Materials and Methods:**

The three reported clinical cases correspond to adult patients who were treated with fixed orthodontics and premolar extractions. Measurements of upper incisor position (UIP), upper incisor inclination (UII), upper lip position (ULP), and upper lip inclination (ULI) were performed on pre-treatment and post-treatment cephalometric radiographs for assessment of changes.

**Results:**

In the first case a variation of -1 mm was found for both the UIP and the ULP, as well as a variation of the UII and the ULI, although in different magnitude. Case 2 presented a 2 mm variation in the UII with minimal changes in the upper lip (∆ ULP = 0 mm and ∆ ULI =-0.5 mm) and in case 3 a 2 mm variation was obtained for both IIS and ILS.

**Conclusions:**

The results obtained in this case reports show us a wide variability, so it is impossible to accurately predict changes in soft tissues as a response to tooth movement.

**Clinical significance:**

Predicting tooth movement changes in soft tissues is critical during the initial planning phase of orthodontic treatment.

## RESUMEN

**Objetivo:**

Determinar los cambios en la posición e inclinación del incisivo y el labio superiores después del tratamiento de ortodoncia en una serie de 3 casos clínicos.

**Materiales y métodos:**

Los tres casos clínicos reportados corresponden a pacientes adultos que fueron tratados con ortodoncia fija y extracciones de premolares. Se realizaron mediciones de la posición de los incisivos superiores (UIP), la inclinación de los incisivos superiores (UII), la posición del labio superior (ULP) y la inclinación del labio superior (ULI) en radiografías cefalométricas previas y posteriores al tratamiento para evaluar los cambios.

**Resultados:**

En el primer caso, se encontró una variación de -1 mm tanto para la UIP como para la ULP, así como una variación de la UII y la ULI, aunque en diferente magnitud. El caso 2 presentó una variación de 2 mm en el UII, con cambios mínimos en el labio superior (∆ ULP = 0 mm y ∆ ULI = -0,5 mm). En el caso 3 se obtuvo una variación de 2 mm tanto para IIS como para ILS.

**Conclusiones:**

Los resultados obtenidos en este reporte de caso nos muestran una amplia variabilidad, por lo que es imposible predecir con precisión los cambios en los tejidos blandos como respuesta al movimiento dentario.

**Importancia clínica:**

Predecir los cambios en el movimiento de los dientes en los tejidos blandos es fundamental durante la fase de planificación inicial del tratamiento de ortodoncia.

## INTRODUCTION

Dental malocclusions (MO) represent one of the most frequent dental alterations ^1^^,^^2^. Today within the goals of orthodontic treatment in addition to the establishment of ideal skeletal and dental relationships is considered facial aesthetics due to the high aesthetic expectations of patients ^3^^-^^6^.

The biomechanics use in orthodontic treatment can generate changes in the underlying soft tissue, so the possibility of predicting these changes is of paramount importance ^7^^-^^9^.

Some research results suggest that tooth movement may not have a proportional effect on the profile contour and that soft tissues can sustain themselves ^10^^-^^12^. On the other hand, there are studies that mention factors that can generate a variation in the final disposition of the soft tissues. Some of them are the position and inclination of the upper incisor, which means that tooth movement in some direction can have an influence on the position and inclination of the upper lip ^13^^-^^15^.

The Published studies showed results as substantial soft tissue changes in orthodontic treatments performed with premolar extractions, obtaining results with predictable aesthetic improvements in patients with initial diagnosis of protrusion ^16^^-^^19^. However, a 2018 study aimed to determine soft tissue changes following orthodontic treatment with and without premolar extractions had similar results in both groups ^20^.

The aim of this work was to determine the changes in the position and inclination of the upper incisor and upper lip after orthodontic treatment presented in the following case reports.

## MATERIALS AND METHODS

The three reported clinical cases correspond to adult patients who were treated at the Dental Clinic of the Universidad Científica del Sur in Lima, Peru by different operators, diagnosed with different types of malocclusions which were treated with fixed orthodontics for a minimum period of 2 years and whose treatment plan estimated the performance of premolar extractions. None had any relevant medical history.

At the end of the orthodontic treatment, the measurements were made corresponding to four radiological characteristics such as the PIS (perpendicular distance between the incisal edge of the central incisor superior to the NA plane), the IIS (angle formed by the major axis of the upper central incisor and by the NA plane), the PLS (perpendicular distance in millimeters between the upper lip and the Spradley line) and the ILS (angle formed by the tangent of the upper lip and the N-PERP trace) both in the cephalometric radiographs of the pre-treatment and post-treatment for subsequent evaluation of the changes that occurred. Informed consent was provided by the patient for publication purposes. The study was reviewed and approved by the Research Ethics Committee of the Científica del Sur University (POS-95-2022-00497).

## CLINICAL CASES

### CLINICAL CASE 1

**Figure 1:**
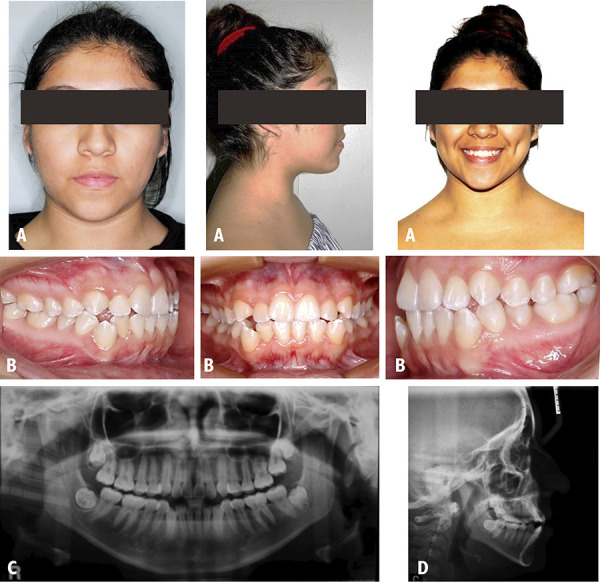
Case 1. Initial pre-treatment records. A: Extraoral photographs. B: Intraoral photographs. C: Panoramic radiograph. D: Cephalometric radiograph.

**Figure 2:**
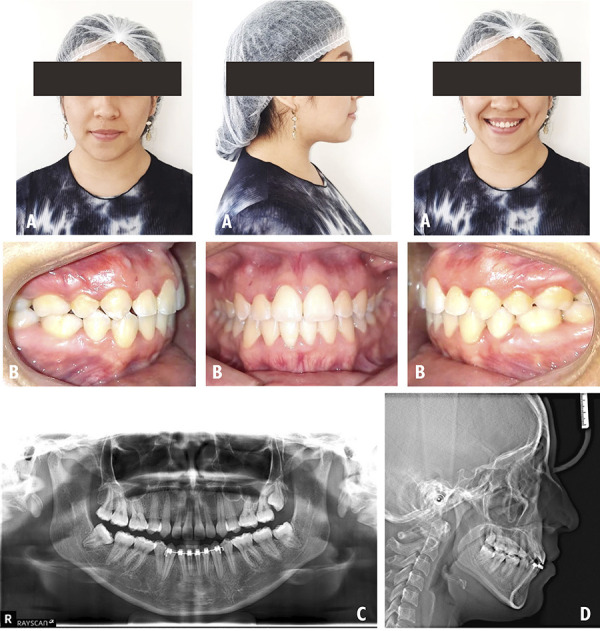
Case 1. Post-treatment records. A: Extraoral photographs. B: Intraoral photographs. C: Panoramic radiograph. D: Cephalometric radiograph.

A Female patient of 22 years who was attended in the clinic for orthodontic consultation, with the main reason: “I want to align my canines”. The extraoral examination revealed to be a mesofacial patient, with a convex profile and competent lips (Fig. 1A). The intraoral examination is classified as a class III MO, with presence of dental crowding, midline deviation and clinical absence of tooth 27 (Fig. 1B). The presence of impacted tooth 27 was confirmed on the panoramic radiograph (Fig. 1C). According to the cephalometric radiography the patient is classified with a Class I skeletal pattern presenting a BNA of 2° (Fig. 1D). Within the objectives, the following points were considered to be corrected: MO class III, impaction of tooth 27, multiple gyroversions and deviation from the midline. The management of orthodontic treatment was carried out in 3 phases: The first phase involved tooth extractions 15, 25, 35 and 45 followed by alignment and leveling. The second phase of treatment was directed to perform space closure. The last phase was the completion phase and was aimed to improve the intercuspation. At the end of the treatment the extraoral examination was performed and her initial features were maintained (Fig. 2A). The intraoral examination shows a class I MO with a deviation of 1 mm from the lower midline to the right side, in addition the clinical presence of tooth 27 is visualized (Fig. 2B). In the panoramic x-ray, the lack of root parallelism of some teeth is still visualized. (Fig. 2 C). On cephalometric radiography the patient maintains ANB of 2° (Fig. 2D). In the evaluation of the changes of the UIP presented a variation of -1 mm, the UII presented a variation of -4°, with respect to the ULP there is a variation of -1 mm and the ULI presented a variation of -0.5° (Table 1).

**Table 1:** Changes in the treatment of the position and inclination of the incisor and upper lip.

N° case	UIP (mm)			UII (°)			ULP (mm)			ULI (°)		
	Pre-tto	Post-tto	∆	Pre-tto	Post-tto	∆	Pre-tto	Post-tto	∆	Pre-tto	Post-tto	∆
Case 1	3	2	-1	29	25	-4	1	0	-1	6	5,5	-0,5
Case 2	-1	-1,5	-0,5	12	14	2	0	0	0	1	0,5	-0,5
Case 3	5,5	5	-0,5	26	28	2	5,5	6	0,5	10	12	2

UIP (Upper incisor position), UII (Upper incisor inclination), ULP (Upper lip position), ULI (Upper lip inclination).

### CLINICAL CASE 2

**Figure 3:**
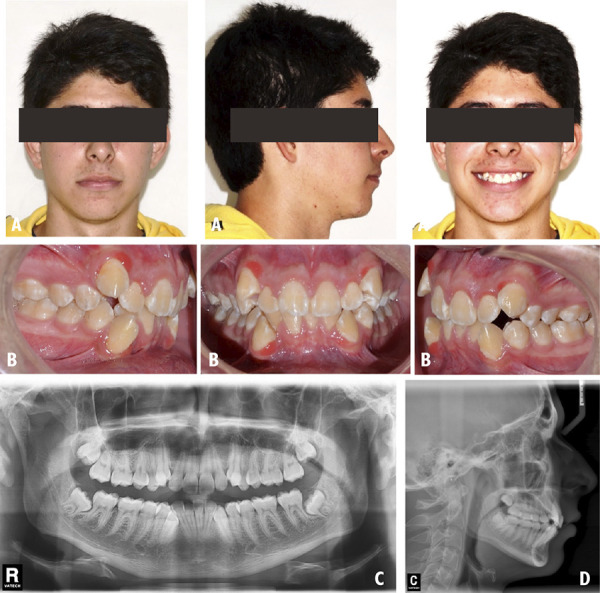
Case 2. Initial pre-treatment records. A: Extraoral photographs. B: Intraoral photographs. C: Panoramic radiograph. D: Cephalometric radiograph.

**Figure 4:**
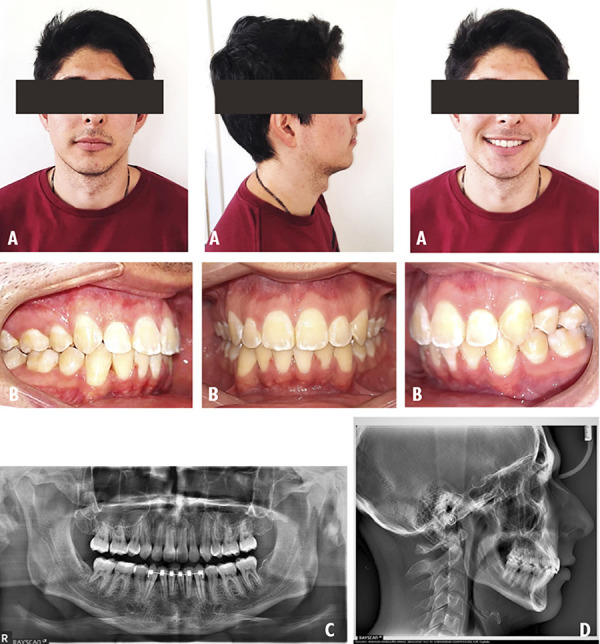
Case 2. Post-treatment records. A: Extraoral photographs. B: Intraoral photographs. C: Panoramic radiograph. D: Cephalometric radiograph.

A 26-year-old male patient who attended the clinic for orthodontic consultation, whose main reason: “Improve my smile”. The extraoral examination revealed a dolicofacial patient with a convex profile and competent lips (Fig. 3A). The intraoral examination classified it as a Class I MO with presence of severe dental crowding and deviation of 2.5 mm from the lower midline to the right side (Fig. 3B). No relevant findings were found on the panoramic radiograph (Fig. 3C). On the cephalometric radiography, the patient was classified with a Class II skeletal pattern with 7° ANB (Fig. 3D). The treatment objectives considered resolving the severe crowding, the Spee curve and multiple gyroversions were considered to be resolved. The management of orthodontic treatment was carried out in 4 phases: In the first phase the teeth 14, 24, 34 and 44 were extracted the anchorage added was a TPA with Nance button in the upper arch and a lingual arch in the lower arch to subsequently continue with the distalization of canines. In the second phase, the alignment and leveling was performed. In the third phase, the closing of spaces was carried out. The last completion phase was aimed at improving intercuspation through the use of intermaxillary elastics (EIM). At the end of the treatment the extraoral examination was performed and the initial features were maintained (Fig. 4A). The intraoral examination shows a class I MO with 0.5 mm deviation from the lower midline to the right side and resolution of the initial gyroversions (Fig. 4B). In the panoramic x-ray, the lack of root parallelism of some teeth is still visualized (Fig. 4C). On cephalometric radiography the patient keeps an ANB of 7° (Fig. 4D). In the evaluation of the UIP presented a variation of -0.5 mm, the UII presented a variation of 2°, with respect to the ULP there is no variation and the ULI presented a variation of -0.5° (Table 1).

### CLINICAL CASE 3

**Figure 5:**
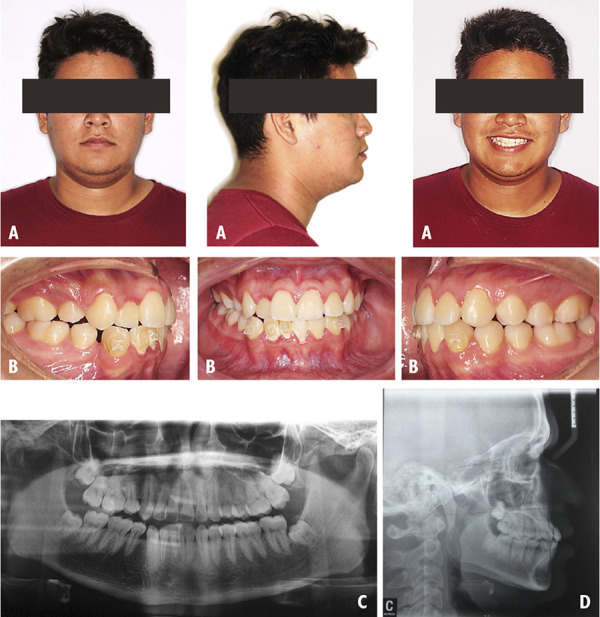
Case 3. Initial pre-treatment records. A: Extraoral photographs. B: Intraoral photographs. C: Panoramic radiograph. D: Cephalometric radiograph.

**Figure 6:**
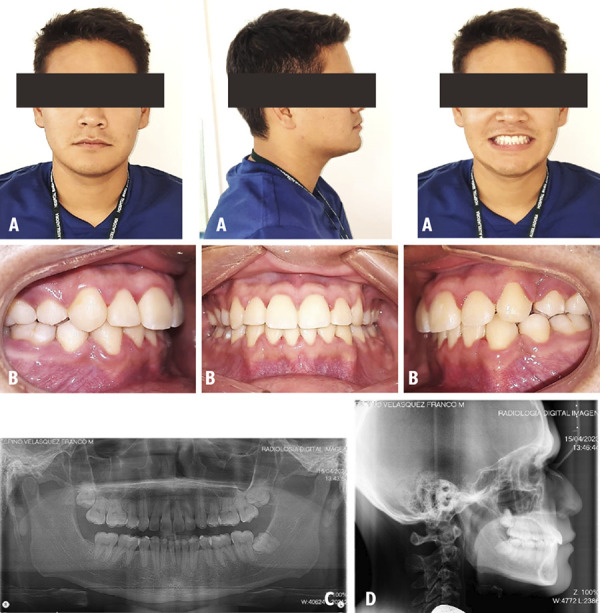
Case 3. Post-treatment records. A: Extraoral photographs. B: Intraoral photographs. C: Panoramic radiograph. D: Cephalometric radiograph.

A 24-year-old male patient who was attended the clinic for orthodontic consultation, the main reason: “I want my teeth aligned”. The patient referred to present an unfinished previous orthodontic treatment. The extraoral examination revealed a mesofacial patient with a convex profile and competent lips (Fig. 5A). The intraoral examination showed a class III MO, mild dental crowding and clinical absence of teeth 14 and 44 (Fig. 5B). The third molars impactation were found on the panoramic radiographic (Fig. 5C). According to the cephalometric radiography, the patient is classified as a class I skeletal pattern with an ANB of 2° (Fig. 5D). The main objective was to correct the class III malocclusion. The management of orthodontic treatment was carried out in 3 phases: The first phase involved tooth extractions 24 and 34 followed by alignment and leveling. The second phase of treatment was directed to perform space closure. The last completion phase was aimed to improve intercuspation through the use of EIM. The extraoral features were maintained at the end of treatment (Fig. 6 A). Intraoral examination is observed a class I MO (Fig. 6B). In the panoramic x-ray, the lack of parallelism of the roots is still visualized (Fig. 6C). On cephalometric radiography the patient maintains the ANB 2° (Fig. 6D). In the evaluation of the UIP presented a variation of -0.5 mm, in the UII presented a variation of 2°, with respect to the ULP a variation of 0.5 mm was found and the ULI presented a variation of 2° (Table 1).

## DISCUSSION

The facial balance and harmony are determined by the interrelation of various factors such as skeletal and soft tissue characteristics, as well as the position and inclination characteristics of the incisors. There are studies that directly relate the effect of orthodontic treatment with the position of the lips as a result of variations in position and inclination of the incisors ^7^^,^^11^.

When analyzing the results obtained, the wide variability between the three clinical cases presented is perceptible, which is similar to what was obtained in a study carried out by Mirabella and Bacconi ^11^ in 2008, who highlighted a marked diversity of results which did not allow the possibility of making a prediction with pressure regarding possible changes in soft tissue.

In the study carried out by Baik and Choi (^7^) in 2022, they concluded that the more the incisor apical root is retracted, there is a greater degree of movement towards the posterior sector compared to the inclination movement, which corresponds to the results obtained in case 1 where the variation of tooth movement in direction and magnitude is -1 mm in the UIP and the ULP. On the other hand, the UII is followed in the direction of movement by the ULI but in a lesser extent (IIS = -4 ° and ILS =-0.5°).

In a systematic review carried out by Leonardi and Annunziata (^17^) in 2010, they mention that the changes in soft tissues caused by tooth movement are small and do not significantly modify the profile even in cases treated with premolar extractions, which is coincident with the 3 clinical cases presented in this report where the variations of the ULP range between 0.5 mm and 1 mm, like in the ULI whose range ranges between 0.5 mm and 2 mm.

Given that the present study is a report of only three cases, it is insufficient to obtain a prediction pattern or proportion, however, there is the possibility that carrying out studies with a larger sample can better visualize the possibility of predicting changes in soft tissues versus the behavior of tooth movement.

A good quality of the images, as well as maintaining an adequate protocol for the radiographic will allow to obtain results of greater reliability and precision.

An important factor to consider in future research is the influence of the thickness of the upper lip given that in a study by El Asmar and Ghoubril of 2020 ^10^ where they determined the impact of extractions on the soft profile, finding that for patients with thick lips the positioning of the incisor did not generate a perception of change in the profile compared to patients who had thin lips.

Likewise, biomechanics must be considered as an important factor given that the type of biomechanics as well as the type of anchor used can influence the degree of tooth movement of the previous sector; undoubtedly, the presentation of this case report can be a precedent for future research.

## CONCLUSIONS

The results obtained in the present study do not allow to obtain the possibility of accurately predicting the changes with respect to the relationship of tooth movement and its impact on soft tissues due to its marked variability.

## Financing:

His research work was self-financed.

## References

[1] Alhammadi MS, Halboub E, Fayed MS, Labib A, El-Saaidi C (2018). Global distribution of malocclusion traits: A systematic review. Dental Press J Orthod.

[2] Lombardo G, Vena F, Negri P, Pagano S, Barilotti C, Paglia L (2020). Worldwide prevalence of malocclusion in the different stages of dentition A systematic review and meta-analysis. Eur J Paediatr Dent.

[3] Aksakalli S, Demir A (2014). Facial soft tissue changes after orthodontic treatment. Niger J Clin Pract.

[4] He D, Gu Y, Sun Y (2020). Correlations between objective measurements and subjective evaluations of facial profile after orthodontic treatment. J Int Med Res.

[5] Luyten J, Vierendeel M, De Roo NMC, Temmerman L, De Pauw GAM (2022). A non-cephalometric three-dimensional appraisal of soft tissue changes by functional appliances in orthodontics: a systematic review and meta-analysis. Eur J Orthod.

[6] Christou T, Betlej A, Aswad N, Ogdon D, Kau CH (2019). Clinical effectiveness of orthodontic treatment on smile esthetics a systematic review. Clin Cosmet Investig Dent.

[7] Baik W, Choi SH, Cha JY, Yu HS, Lee KJ (2022). Comparison of soft tissue changes between incisor tipping and translation after premolar extraction. Korean J Orthod.

[8] Zhang X, Mei L, Yan X, Wei J, Li Y, Li H (2019). Accuracy of computer-aided prediction in soft tissue changes after orthodontic treatment. Am J Orthod Dentofacial Orthop.

[9] Konstantonis D, Vasileiou D, Papageorgiou SN, Eliades T (2018). Soft tissue changes following extraction vs nonextraction orthodontic fixed appliance treatment: a systematic review and meta-analysis. Eur J Oral Sci.

[10] El Asmar R, Akl R, Ghoubril J, El Khoury E (2020). Evaluation of the ideal position of the maxillary incisor relative to upper lip thickness. Am J Orthod Dentofacial Orthop.

[11] Mirabella D, Bacconi S, Gracco A, Lombardo L, Siciliani G (2008). Upper lip changes correlated with maxillary incisor movement in 65 orthodontically treated adult patients. World J Orthod.

[12] (2002). The relationship between lip thickness and lip displacement in response to incisor movement. American Journal of Orthodontics and Dentofacial Orthopedics.

[13] Shen T, Shi H (2021). Relationship between the position of the maxillary incisor and upper lip. Am J Orthod Dentofacial Orthop.

[14] Huang J, Li CY, Jiang JH (2018). Facial soft tissue changes after nonsurgical rapid maxillary expansion a systematic review and meta-analysis. Head Face Med.

[15] Liu Y, Yang ZJ, Zhou J, Xiong P, Wang Q, Yang Y (2019). Soft Tissue Changes in Patients With Dentoalveolar Protrusion Treated With Maximum Anchorage A Systematic Review and Meta-analysis. J Evid Based Dent Pract.

[16] (1997). The effect of premolar extractions on the soft-tissue profile in adult African American females. The Angle Orthodontist.

[17] Leonardi R, Annunziata A, Licciardello V, Barbato E (2010). Soft Tissue Changes Following the Extraction of Premolars in Nongrowing Patients With Bimaxillary Protrusion A Systematic Review. The Angle Orthodontist.

[18] Scott Conley R, Jernigan C (2006). Soft Tissue Changes after Upper Premolar Extraction in Class II Camouflage Therapy. The Angle Orthodontist.

[19] Au J, Mei L, Bennani F, Kang A, Farella M (2019). Three-dimensional Analysis of Lip Changes in Response To Simulated Maxillary Incisor Advancement. The Angle Orthodontist.

[20] Janson G, Castello Branco N, Aliaga-Del Castillo A, Henriques JFC, de Morais JF (2018). Soft tissue treatment changes with fixed functional appliances and with maxillary premolar extraction in Class II division 1 malocclusion patients. European Journal of Orthodontics.

